# Association of telehealth use with endocrine therapy adherence, metastasis incidence and healthcare costs in breast cancer: A claims-based cohort study

**DOI:** 10.1007/s11764-025-01950-x

**Published:** 2025-12-19

**Authors:** Shaimaa Elshafie, Lorenzo Villa Zapata

**Affiliations:** Department of Clinical and Administrative Pharmacy, College of Pharmacy, University of Georgia, Athens, GA USA

**Keywords:** Telehealth, Breast cancer, Endocrine therapy, Adherence, Costs, Metastasis, Policy

## Abstract

**Purpose:**

The expansion of digital health offers a promising avenue to improve care delivery and optimize treatment outcomes. This study evaluated telehealth utilization and its impact on endocrine therapy adherence, clinical outcomes, and healthcare costs.

**Methods:**

This retrospective cohort study used claims data from the Merative MarketScan database. Eligible patients were females under 65 years old who were diagnosed with nonmetastatic breast cancer in 2018 and maintained commercial insurance for one year pre-diagnosis and five years post-endocrine therapy initiation. Telehealth utilization was assessed as a binary (user vs. nonuser) measure. Primary outcomes were endocrine therapy adherence, metastasis incidence, and patient-incurred healthcare costs. Associations between telehealth and outcomes were tested using generalized linear models.

**Results:**

Among 1,141 eligible patients (mean age: 51 years), 874 (77%) used telehealth, totaling 8,350 visits over five years. Telehealth utilization was consistent across age groups and insurance plans but varied significantly by geographic region, urban–rural status, comorbidity burden, and endocrine therapy regimen. Telehealth use was significantly associated with better adherence (adjusted odds ratio = 1.58; 95% CI: 1.31–1.91; *p* < 0.0001) but not with metastasis rates. Notably, telehealth users incurred 15% higher out-of-pocket medical costs compared to nonusers (*p* < 0.0001), though prescription costs did not differ significantly.

**Conclusions:**

Findings demonstrate telehealth benefits for long-term adherence to endocrine therapy among commercially insured women under 65 with breast cancer.

**Implications for Cancer Survivors:**

Telehealth may help breast cancer survivors stay adherent to endocrine therapy, but its financial burden could limit accessibility and equity in oncology care.

**Supplementary Information:**

The online version contains supplementary material available at https://doi.org/10.1007/s11764-025-01950-x.

## Background

Breast cancer is the most prevalent cancer among women worldwide [1], accounting for approximately one-third of new cancer diagnoses in the United States (US) annually [2]. While early stages of the disease are often curable with appropriate therapeutic interventions, long-term management is critical for reducing the risk of cancer recurrence. Endocrine therapy (ET) has been a cornerstone treatment for hormone receptor-positive (HR +) breast cancer which comprises the majority of cases [3]. Adherence to ET over extended periods (5–10 years) is essential for optimizing disease-free survival outcomes [4]. However, adherence rates remain suboptimal, particularly among young women, due to multiple factors such as treatment-related side effects, financial burden, and healthcare access limitations [5–8].

Addressing barriers to adherence requires effective strategies that carefully consider cost implications, given the financial toxicity of cancer care. Breast cancer management has the highest national medical expenditures among all cancer types at annual costs exceeding $29 billion [9]. Patients with cancer endure significant costs for medical services, diagnostic procedures, pharmaceutical medications, supportive care and indirect expenses (e.g., lost productivity, extra childcare during treatment) [10]. Financial distress is more paramount among patients under 65 who are ineligible for public insurance coverage and therefore bear high out-of-pocket (OOP) expenses [11, 12]. Additional challenges, such as cancer-related work absenteeism and competing obligations further strain their ability to afford care [12, 13].

The rapid expansion of digital health, especially since the COVID-19 pandemic, presents a promising platform to improve care delivery, overcome treatment barriers, and potentially minimize costs [14]. Telehealth, defined broadly as the remote delivery of healthcare services through telecommunication technologies [15], facilitates timely access to providers, enhances patient engagement, and mitigates logistical and structural barriers to care [16]. Although previous research has examined the role of telehealth in chronic disease management, limited evidence exists on its impact on ET adherence and associated clinical and economic outcomes in patients with breast cancer.

This study assesses the real-world utilization of telehealth services among commercially insured women under 65 with nonmetastatic breast cancer and evaluates its association with ET adherence, metastasis incidence, and overall patient-incurred healthcare costs. We hypothesize that telehealth use correlates with improved medication adherence and lower long-term healthcare costs due to better disease control. By leveraging large claims data from the Merative MarketScan database, this study provides valuable insights into the potential benefits and limitations of telehealth in oncology care, particularly for vulnerable populations.

## Methods

### Data source

This retrospective cohort study includes data from the Merative MarketScan database, which captures healthcare utilization and cost data for a significant portion of privately insured individuals in the US. The database provides comprehensive real-world information on clinical services, procedures, prescribed treatments along with their associated financial elements. Although the claims data are fully de-identified, the study protocol was submitted to the Institutional Review Board of the University of Georgia which deemed the research activity exempt from review requirements.

### Study design

Following the Strengthening the Reporting of Observational Studies in Epidemiology (STROBE) guidelines [17], we examined the association between telehealth utilization and three outcomes: adherence to ET, metastasis development, and direct OOP healthcare costs within a commercially insured population in the US. To minimize inherent biases in nonrandomized observational studies, we applied a new-user design [18], a rigorous approach that excludes prevalent ET users and focuses on individuals initiating therapy for the first time.

**Fig. 1:**
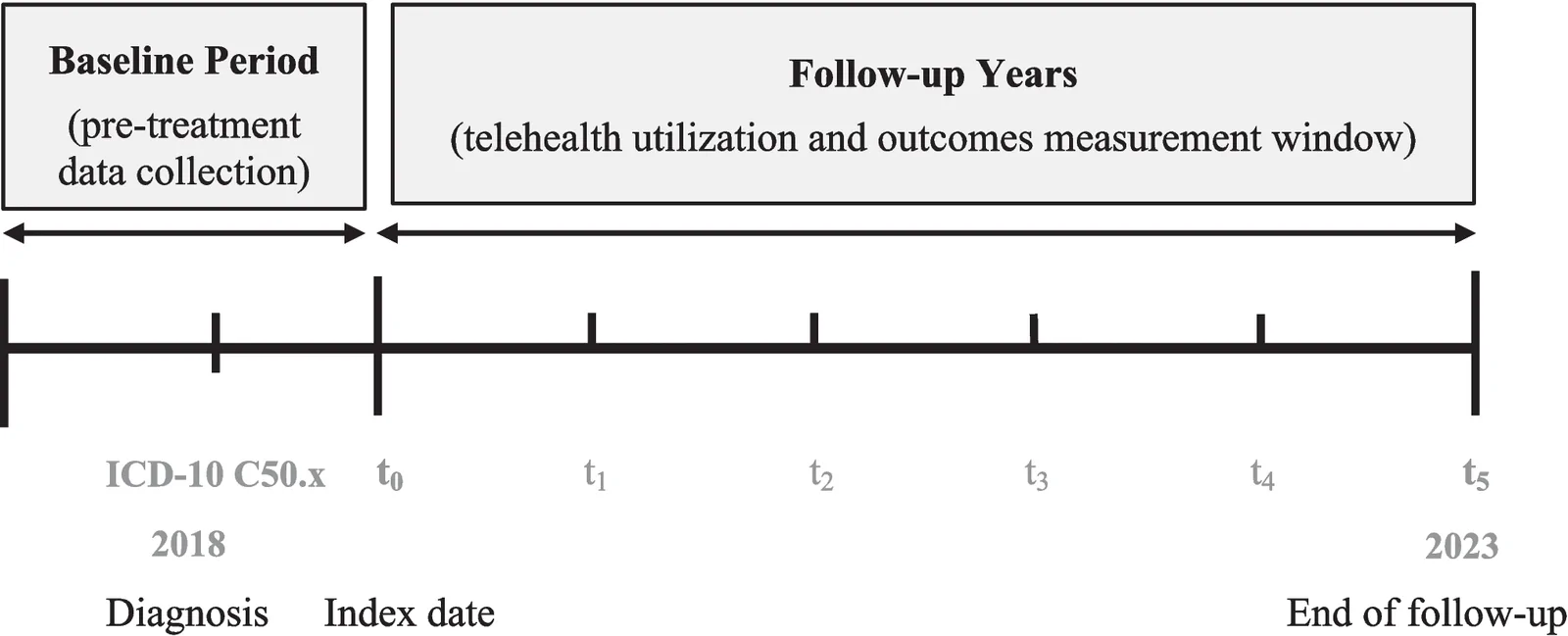
Schematic diagram of the study design and timeline, 2017–2023

Women under age 65 who initiated ET (tamoxifen, letrozole, anastrozole, or exemestane) following a breast cancer diagnosis in 2018 were followed for five years. The index date was defined as the date of the first ET prescription. Outcomes were evaluated from the index date until the end of the follow-up period and annually to account for temporal trends. All covariates were measured at or before the index date. A schematic diagram illustrating the study design is provided in Figure 1.

### Study population

We included females under the age of 65 with commercial insurance who were newly treated with ET after a primary breast cancer diagnosis in 2018 (ICD-10-CM: C50.x). Incident ET users were defined as patients with no previous claims for ET. The study cohort included women who were prescribed a single ET regimen as well as those who switched regimens during the study period. Treatment exposure was determined using National Drug Codes (NDCs) in dispensed prescription claims (Supplementary Table 1).

Eligibility required continuous insurance enrollment for: (a) one year prior to diagnosis to establish treatment history and baseline health status, and (b) five years after ET initiation to enable longitudinal follow-up. Women with metastatic disease at the time of cancer diagnosis or prior to ET initiation were excluded. Also, patients who developed metastases within three months of treatment initiation were excluded to minimize potential misclassification arising from delays in clinical data reporting inherent to administrative claims data. The presence of ICD-10-CM codes for secondary malignancies (C77.x, C78.x, C79.x, C80.0) was used as evidence of metastasis.

### Variables definition

Telehealth was defined as healthcare services delivered remotely through any telecommunication technology regardless of the setting. Telehealth utilization was identified via corresponding procedure codes, modifiers, and place of service codes. We created a dichotomous variable to classify patients as either telehealth users or nonusers as well as a continuous variable to measure the total number of visits.

Outcomes included adherence to ET, metastasis incidence, and the total direct healthcare payments incurred by patients. Adherence was measured by the Proportion of Days Covered (PDC) which ranges from 0 to 100%, with higher values indicating consistent prescription refills and better adherence. In alignment with established thresholds in prior research, women were considered adherent if their PDC was ≥ 80%. Metastasis outcomes were defined as an incident diagnosis of secondary malignancy during the follow-up period. Cost included patient-level expenses for deductibles, copayments, and coinsurance associated with outpatient services, hospitalizations, and prescription medications. All costs were inflation-adjusted to 2023 US dollars (USD) using the Consumer Price Index from the US Bureau of Labor Statistics website [19].

Covariates included patient’s age group, geographic region, rurality status (rural if the Metropolitan Statistical Area [MSA] value was zero), insurance plan type, and ET regimen received. Comorbidity burden was measured using the Charlson Comorbidity Index (CCI). The CCI assigns weighted scores to preexisting comorbid conditions based on their associated mortality risk, with higher scores indicating a greater comorbidity burden and worse prognosis [20, 21]. Estrogen receptor status was identified using ICD-10-CM codes Z17.0 (positive status) and Z17.1 (negative status), but due to underreporting of these codes in claims data, this variable was used only descriptively. A detailed description of all variables, including their definitions and categorization is provided in Supplementary Table 2.

### Statistical analyses

We used descriptive statistics to summarize cohort characteristics, reporting means and standard deviations (SDs) for continuous variables and frequencies and percentages for categorical variables. Differences between telehealth users and nonusers were assessed using bivariate analyses, including chi-square, t-tests, and Wilcoxon rank-sum tests.

Telehealth utilization, adherence, metastasis incidence, and OOP costs were evaluated over five years and annually. For binary outcomes, generalized linear mixed-effects models (GLMMs) with a logistic link were used to estimate odds ratios (ORs) along with their corresponding 95% confidence intervals (CIs). For continuous outcomes, generalized linear models (GLMs) with a Gamma distribution and log link were used to estimate cost ratios and their 95% CIs, with results reported as percent change in cost for interpretability.

Model selection was guided by the nature and distribution of each outcome variable. Random effects accounted for within-subject correlations across repeated measures, and fixed effects included demographic and clinical covariates as well as the observation year. The null hypotheses tested whether the risks of nonadherence, metastasis incidence, and average OOP costs were equivalent between groups over time. All analyses were performed using SAS (version 9.4 TS1M6, SAS Institute, Cary, NC). Statistical significance was defined as a p-value of ≤ 0.05.

## Results

### Patient selection

**Fig. 2:**
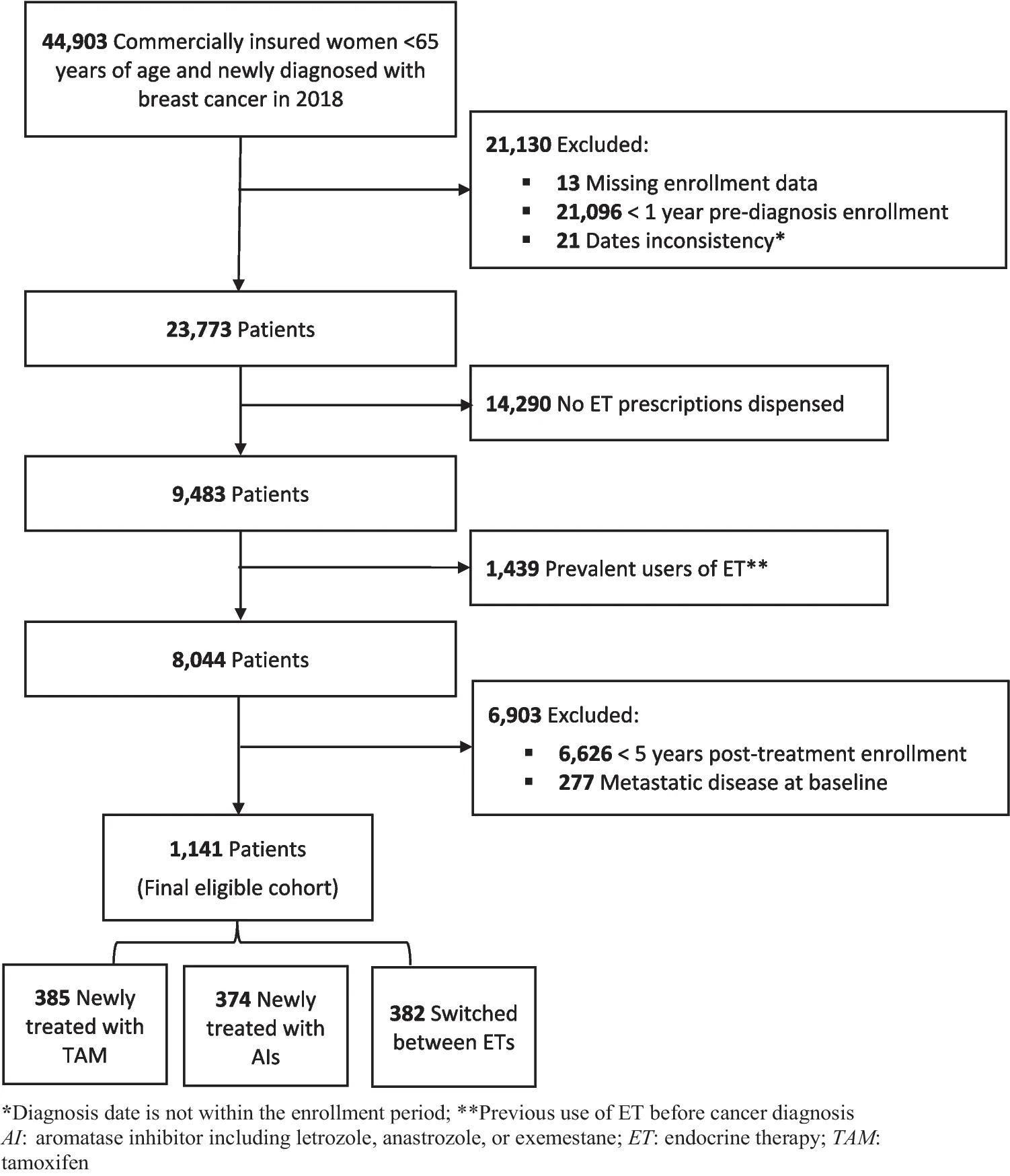
Flowchart of patients included in the study cohort

A search of the administrative commercial claims database identified over 44,000 women under the age of 65 with a breast cancer diagnosis in 2018. After excluding patients without continuous insurance enrollment or without dispensed ET prescriptions, 9,483 patients remained. Of these, 8,044 were new ET users (i.e., no prior prescriptions). We further excluded patients who were not continuously enrolled for five years after ET initiation (*n* = 6,626) and those with metastatic disease before, at, or within three months of treatment initiation (*n* = 277). The final cohort consisted of 1,141 patients who met all eligibility criteria. Figure 2 outlines the cohort selection process.

### Cohort characteristics

Table 1 summarizes the demographic and clinical characteristics of the 1,141 women included in the study. The mean age was 51 years (SD: 6 years) with over half aged 45 to 54. Most patients were in southern states (*n* = 497; 44%), and a small proportion resided in rural areas (*n* = 113; 10%). The most common insurance plan was preferred provider organization (*n* = 512; 46%). Patients exhibited a moderate burden of comorbidities with 92% having a CCI score of 2 or 3.

**Table 1:** Demographic and baseline clinical characteristics of the study cohort

**Socio Demographics**	**Total (*N*** **= 1,141)**
Age at diagnosis	
Mean years ± SD	51 ± 6
Median years (IQR)	52 (47–56)
Age group	
< 35	11 (1)
35–44	133 (12)
45–54	609 (53)
55–64	388 (34)
Region	
Northeast	197 (17)
North Central	284 (25)
South	497 (44)
West	161 (14)
Rurality	
Urban	885 (78)
Rural	113 (10)
Unknown	143 (12)
Plan type	
PPO	512 (46)
HMO	189 (17)
CDHP	169 (15)
HDHP	141 (13)
Others	113 (9)
**Clinical Factors**	
Comorbidity (CCI score)	
Mean score ± SD	2 ± 1
0–1	0 (0)
2–3	1,055 (92)
> 3	86 (8)
Estrogen receptor status	
Positive	1,049 (92)
Negative	20 (2)
Unknown	72 (6)
Endocrine treatment	
TAM	385 (34)
AIs [LET, ANA, EXE]	374 [89, 274, 11] (33)
Switchers	382 (33)

All values are expressed as numbers (percentages) unless otherwise noted

*AI *aromatase inhibitor, *ANA *anastrozole, *CCI *Charlson Comorbidity Index, *CDHP *Consumer-Driven Health Plan, *EXE *exemestane, *HDHP *High-Deductible Health Plan, *HMO *Health Maintenance Organization, *IQR *interquartile range, *LET *letrozole, *PPO *Preferred Provider Organization, *SD *standard deviation, *TAM *tamoxifen

All patients initiated ET in the same year as their cancer diagnosis. The majority of tumors (92%) were estrogen receptor positive. One-third of the patients (*n* = 385) received tamoxifen alone, another third (*n* = 374) were prescribed a single aromatase inhibitor (primarily anastrozole), and the remaining 33% switched between regimens during follow-up.

### Telehealth utilization and five-year outcomes

During the five-year follow-up, 77% of the cohort (874 of 1,141 patients) utilized telehealth services at least once, accounting for 8,350 total visits. Age and insurance plan type were comparable between telehealth users and nonusers. However, telehealth use varied significantly by region (*p* = 0.0064) with the highest prevalence in the West (86%) and the lowest in the North Central region (71%). Urban residents used telehealth more than rural residents (78% vs. 70%, *p* = 0.0480). Telehealth use was also more likely among patients with higher comorbidity scores (*p* = 0.0073) and those who switched ET regimens (*p* = 0.0013).

By year five, only half of the cohort remained adherent to ET. The average PDC over five years was 76% indicating that patients had access to their medication on approximately 3 out of every 4 days. Among the 1,141 patients, 57 (5%) developed metastasis within five years with an average time to metastasis of 2.3 years (SD: 1.3 years). All-cause hospitalization occurred in 153 patients (13%) with an average hospital stay of four days.

Total medical expenditures over the five-year period exceeded $8.5 million averaging $7,510 per patient (SD: $5,782). Prescription spending amounted to over $2.1 million ($1,854 per patient), with ET accounting for 9% of these costs. Table 2 provides a summary of cohort measures over the follow-up period.

**Table 2:** Summary of study cohort measures over the five-year follow-up

Measure	Mean ± SD	Total (n)	Percentage (%)^a^
Telehealth Utilization			
User (≥ 1 visit)	-	874	77%
Nonuser	-	267	23%
Total visits (count)	7 ± 18	8,350	-
Adherence Status			
Adherent	-	571	50%
Nonadherent	-	570	50%
Adherence rate (PDC)	76% ± 39% ^b^		-
Clinical Outcomes			
Did not develop metastasis	-	1,076	95%
Developed metastasis	-	57	5%
Time to metastasis (years)	2.3 ± 1.3	-	-
Hospitalized (≥ 1) for any reason	-	153	13%
Length of Stay (days)	4 ± 4	853	-
Cost Summary (USD)			
Outpatient medical costs	$7,312 ± $5,600	$8,342,545	-
Inpatient medical costs	$830 ± $1,914	$226,622	-
Total medical costs	$7,510 ± $5,782	$8,569,166	-
Total prescription costs	$1,854 ± $3,738	$2,115,132	-
Endocrine prescription costs	$171 ± $317	$195,570	-

*PDC *Proportion of Days Covered, *SD *standard deviation, *USD *United States dollar

^a^Percentages are calculated based on the total cohort size (*N* = 1,141)

^b^Percentages may exceed 100% when the total days of drug supply (numerator) surpass the number of days in the observation period (denominator), such as when patients refill prescriptions early

### Assessment of temporal trends

**Fig. 3:**
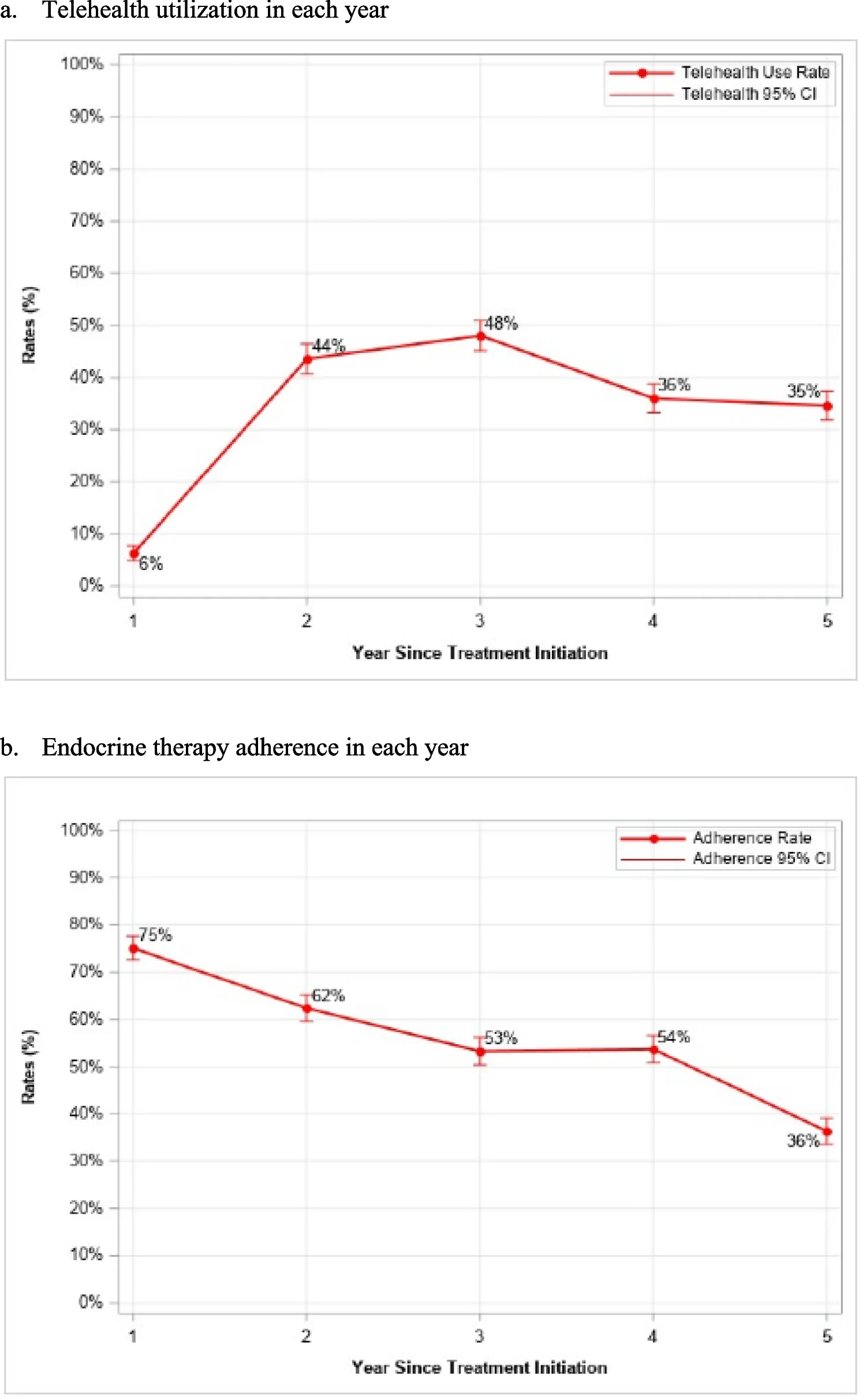

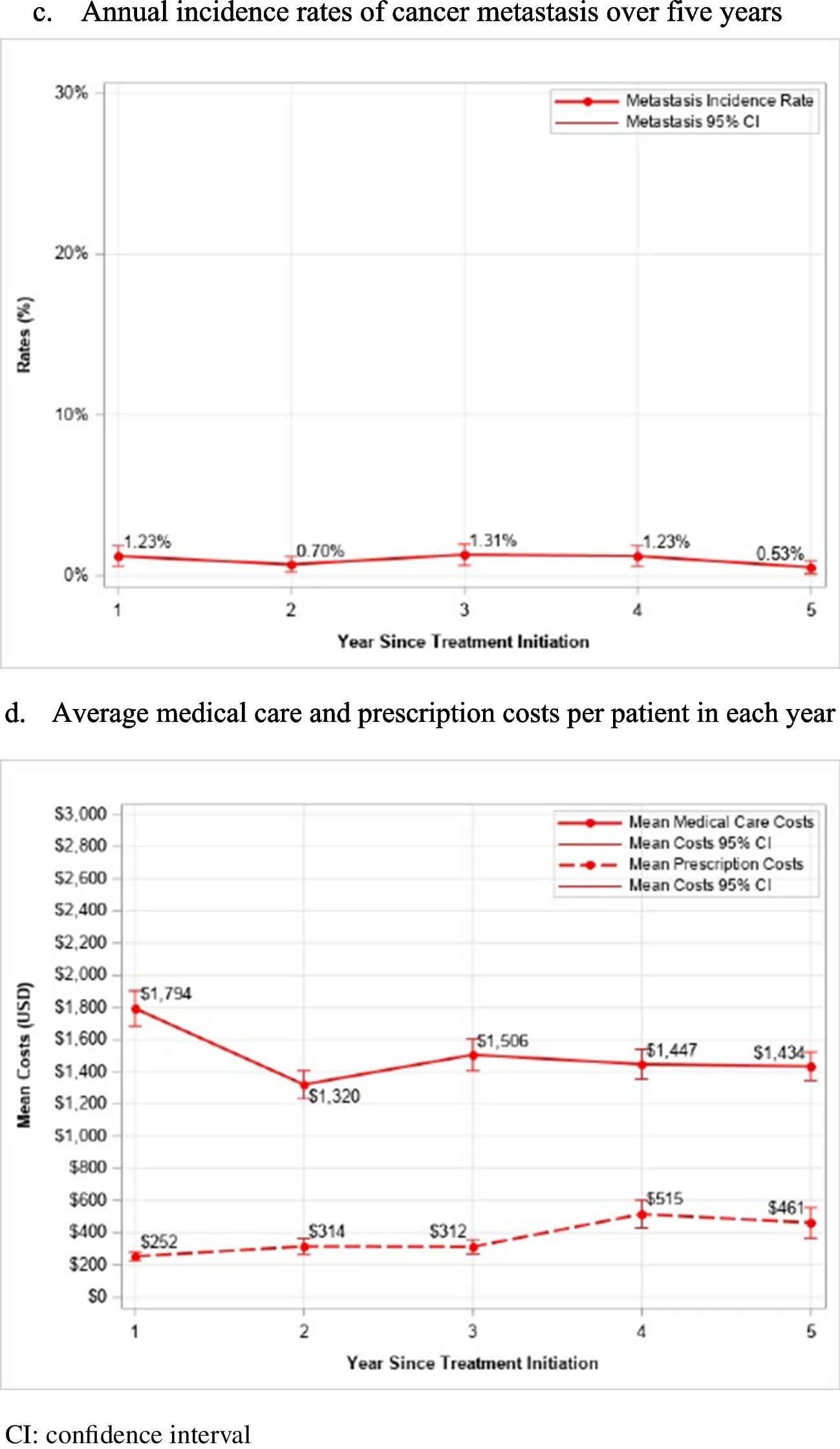
Trends in telehealth utilization, endocrine therapy adherence, cancer metastasis, and healthcare costs over time

Figure 3a–d shows the evolving patterns of telehealth utilization, ET adherence, metastasis incidence, and healthcare costs. Telehealth use increased sharply, rising nearly sevenfold during the second year of ET. This surge coincided with the onset of the COVID-19 pandemic in 2020 (see Supplementary Table 3). Utilization peaked in the third year with over 48% of patients using telehealth services, followed by a slight decline in subsequent years.

Adherence steadily declined over time, from 75% in year one to 36% in year five. Metastasis rates remained low and relatively stable throughout the study period. Average medical care expenditures decreased from $1,794 in year one to $1,320 in year two, then modestly increased and stabilized thereafter. In contrast, average prescription costs per patient increased consistently, fluctuating between $252 and $515 annually.

Statistical testing showed a strong and significant downward trend in adherence (*p* = 0.0092) with time since ET initiation explaining 92% of its variance. On average, adherence proportions decreased by approximately 9% as time progresses. The trend for prescription costs revealed marginal significance (*p* = 0.0502), increasing by approximately $62 annually. Other measures did not show significant temporal changes.

### Assessment of relationships

Telehealth utilization was significantly associated with increased ET adherence in both the baseline model and after adjusting for temporal tends and patient characteristics (OR = 1.58; 95% CI: 1.31–1.91; *p* < 0.0001). The odds of adherence were the highest in year one and declined significantly over time. Other adherence predictors included geographic region, insurance plan type, and ET regimen, with the lowest adherence observed among tamoxifen users. Age, rurality and comorbidity burden were not significantly associated with adherence.

No significant relationship was observed between telehealth utilization and metastasis incidence (OR = 1.54; 95% CI: 0.69–3.47; *p* = 0.2961). However, telehealth use was significantly associated with increased OOP medical care costs, with users spending 15% more than nonusers (95% CI: 9%–22%; *p* < 0.0001). Costs were the highest in year one, the lowest in year two, and relatively stable from years three through five. Geographic region and insurance plan type were also significant cost predictors. Notably, telehealth utilization was not associated with differences in prescription costs.

## Discussion

Our findings demonstrate that telehealth utilization among women under 65 with breast cancer increased during the COVID-19 pandemic and remained an integral part of their care. However, notable variations were observed based on geographic location, urban–rural status, comorbidity burden, and ET regimen. Telehealth use was significantly associated with improved adherence to ET but was also linked to higher long-term OOP costs.

The increased telehealth usage among patients with higher comorbidities and those who switched treatments aligns with previous research [22], and reflects the convenience of remote care for managing complex health conditions and facilitating additional follow-up or monitoring. However, several challenges may hinder broader telehealth adoption within the US healthcare system, including patient and provider preferences, reimbursement policies, limited clinician training in telehealth practices, and inconsistent access to digital technology [23, 24].

Despite the general decline in ET adherence over time, telehealth users had higher odds of maintaining adherence throughout the five-year follow-up period. These results highlight the potential of telehealth to promote medication persistence and are consistent with previous studies demonstrating improved chronic disease management and enhanced medication adherence through digital and telecommunication platforms. A systematic review of 13 studies showed evidence on the effectiveness of telehealth in increasing medication adherence across various chronic conditions [25]. Similarly, recent meta-analyses revealed the effectiveness of telehealth in enhancing medication adherence among patients with diabetes and stroke [26, 27].

We did not find a significant relationship between telehealth utilization and reduced metastasis incidence. The sustainable overall low rates of metastasis and hospitalizations suggest effective disease management within the studied cohort. This finding implies that while telehealth supports adherence, it may not be directly associated with disease progression within the observed timeframe. Additional factors such as cancer biology, treatment response, and broader healthcare access disparities may have a more substantial role in determining clinical outcomes [28].

Despite its positive impact on adherence, telehealth users incurred higher OOP costs compared to nonusers, primarily due to increased expenses for medical services. One possible explanation for these higher costs is the healthy user bias [29], which suggests that patients who are generally more proactive in seeking and utilizing healthcare services are also more likely to adopt telehealth. A recent study of Medicare beneficiaries concluded that the higher costs observed among telehealth users were likely attributable to greater underlying care needs rather than being a direct consequence of telehealth utilization [30]. Alternatively, telehealth itself may be an independent driver of increased healthcare utilization and associated costs. However, Patel et al. reported an average cost savings of $186 per telehealth visit among 11,688 patients with cancer and mainly private insurance in Florida over a 15-month period [31]. These contrasting findings underscore the complexity of telehealth’s financial implications which may vary based on healthcare policies, insurance coverage, and service utilization patterns.

Other studies have highlighted telehealth’s potential for cost savings through various mechanisms, including reduced travel expenses, increased care efficiency, fewer unnecessary specialist referrals, and lower rates of hospitalizations and emergency department visits [32]. These benefits suggest that while telehealth may lead to higher direct medical costs in some cases, its broader economic impact could result in overall healthcare savings, particularly when indirect costs and system-wide efficiencies are considered. Policy efforts should focus on optimizing telehealth accessibility while minimizing the financial burden of care delivery for patients.

This study is the first to use large-scale, real-world data to evaluate telehealth utilization and its long-term clinical and economic implications in this population, which is particularly vulnerable to treatment nonadherence and financial distress. The study offers important insights for clinicians, policymakers, and insurers emphasizing the benefits of telehealth integration in enhancing treatment adherence while also highlighting the need for cost-saving strategies to ensure equitable access. The combined year-by-year and overall study period analysis approach captured both temporal and longitudinal trends in telehealth utilization, adherence patterns, clinical outcomes, and financial impacts. This methodology also minimized confounding effects from external factors, such as healthcare policy changes during the COVID-19 pandemic and variations in telehealth reimbursement policies, while aligning adherence measurements more precisely with individual treatment timelines rather than calendar years.

Further research is warranted to explore patient-reported experiences, satisfaction, quality of life outcomes and the overall cost-effectiveness of telehealth in oncology care. Future studies should examine the impact of telehealth expansion in rural and underserved populations where structural barriers to traditional care are more pronounced.

This study has several limitations. The cohort was restricted to commercially insured patients under 65, so results may not be generalizable to individuals 65 and older or those with public insurance. Additionally, the requirement for continuous insurance coverage for at least six years limits applicability of the findings to populations with interrupted coverage, lower socioeconomic status, no insurance, or those transitioning between insurance types who often have less stable healthcare access and higher financial barriers. The exclusion of these patients may have led to an overestimation of the observed association between telehealth use and adherence. Also, claims data do not provide information on the purpose or content of telehealth visits (e.g., side effect management or proactive care seeking), which limits interpretation of their clinical context. Furthermore, the adherence measurement method by prescription refills may not accurately reflect actual medication consumption. Additionally, our cost estimations were limited to direct healthcare expenditures and did not include indirect costs or potential savings. Finally, cancer–specific variables such as surgical procedures and receipt of adjuvant therapies were not examined.

In conclusion, telehealth utilization among patients under 65 with breast cancer varied by geographic, clinical, and treatment-related factors over the five-year follow-up period. Telehealth emerged as a valuable tool for supporting long-term adherence to endocrine treatment in this commercially insured population, but it did not significantly influence metastasis rates. As digital health continues to evolve, future efforts should focus on refining implementation strategies to maximize patient benefits, address affordability challenges, and ensure equitable access to telehealth services.

## Supplementary Information

Below is the link to the electronic supplementary material.

Supplementary file1 (DOCX 48 KB)

## Data Availability

The datasets generated during and/or analyzed during the current study are not available for sharing due to commercial restrictions.
