# Supplementary material for: Association of telehealth use with endocrine therapy adherence, metastasis incidence and healthcare costs in breast cancer: A claims-based cohort study
Source: J Cancer Surviv. 2025 Dec 19;20(5):2532–42. doi: 10.1007/s11764-025-01950-x (PMC13723652; doi:10.1007/s11764-025-01950-x)
Supplement: Supplementary file 1 [file 11764_2025_1950_MOESM1_ESM.docx]

**Supplementary Appendix**

**Supplementary Table 1: National Drug Codes for Endocrine Therapies**

| **Endocrine Therapy** | **National Drug Code** |
| --- | --- |
| Tamoxifen | 54483121, 54483126, 54483413, 54483422, 54883125, 54883425, 93078201, 93078205, 93078210, 93078256, 93078405, 93078406, 93078410, 93078486, 172565649, 172565658, 172565670, 172565680, 172565746, 172565760, 172565770, 172565780, 310060018, 310060025, 310060060, 310060075, 310060412, 310060430, 310060490, 310073060, 310073130, 378014405, 378014491, 378027401, 378027493, 403150571, 555044603, 555044605, 555044609, 555044663, 555090401, 555090405, 555090414, 591223218, 591223260, 591223319, 591223330, 591247218, 591247260, 591247319, 591247330, 13632012301, 38779034101, 38779034103, 38779034104, 38779034105, 38779034108, 38779034110, 38779034130, 38779034140, 38779034150, 38779034180, 42254034390, 49452757101, 49452757102, 49452757103, 49452757104, 49452757105, 49452757106, 49452775301, 49452775302, 49452775303, 49452775304, 49452775305, 50090094200, 50090253301, 51552083802, 51862044601, 51862044605, 51862044610, 51862044630, 51862044705, 51862044710, 51862044718, 51862044760, 51862044918, 51862044960, 51862045030, 51862045090, 51927297600, 51927507600, 52372075601, 52372075602, 54569038200, 54569038202, 54569376500, 54569376501, 54569571600, 54569585700, 54569853100, 54569860200, 54868300400, 54868300401, 54868300402, 54868300403, 54868300404, 54868300405, 54868428700, 54868428701, 54868428702, 54868428703, 54868428704, 55175550006, 55289058530, 57866661501, 57866661801, 58016065760, 60346004832, 60346090060, 60429090960, 60429091030, 62991115101, 62991115102, 62991115103, 62991115104, 63275986502, 63275986504, 63275986505, 63275986509, 63307047001, 63307047005, 63370025110, 63370025115, 63370025125, 63370025135, 63739026901, 63739026910, 63739026915, 63739026942, 66105083201, 66105083203, 66105083206, 66105083209, 66105083210, 68084092411, 68084092421, 68084093511, 68084093521, 68258596006, 68382082614, 68382082701, 68382082706, 75840011001, 89141012301 |
| Letrozole | 54026913, 78024915, 78088150, 93762056, 378207105, 378207193, 603418016, 16729003410, 16729003415, 35356040930, 42254024330, 42291037490, 50268047611, 50268047615, 51991075910, 51991075933, 53217010830, 54569571400, 54868415100, 54868625200, 55111064630, 59651018030, 59651018090, 60505325503, 60505325508, 62756051183, 63323077230, 68084080311, 68084080321, 68258595503 |
| Anastrozole | 54016413, 93753656, 310020130, 310020137, 378603405, 378603477, 378603493, 395815735, 395815737, 781535631, 904619546, 904622961, 12280034630, 16571042103, 16729003510, 16729003515, 16729003516, 21695099030, 33261095700, 33261095730, 33261095760, 33261095790, 35356027030, 38779227403, 38779227404, 38779227406, 38779255503, 38779255504, 38779255506, 42043018003, 42254016130, 42291008530, 42291008590, 42291010530, 43063038306, 43063038315, 46144020001, 46144020005, 50090245300, 50268007511, 50268007515, 51079032301, 51079032306, 51655063853, 51927443500, 51991062010, 51991062033, 54569573100, 54569619800, 54868500000, 54868613000, 54868613001, 55111064730, 55175550503, 58597808001, 58597808002, 58597808004, 59651023630, 59651023690, 60258086603, 60429028630, 60429028690, 60505298503, 60687011211, 60687011221, 62033037606, 62559067030, 62756025013, 62756025083, 63275993001, 63275993002, 63275993003, 63275993004, 63275993005, 63323012930, 66336053330, 66336053390, 66435041530, 67877017110, 67877017130, 68001015504, 68001015508, 68084044821, 68382020906, 68382020910, 72789000815, 72789000890, 76418001301, 76519122403 |
| Exemestane | 9766304, 54008013, 378500193, 832059530, 47781010830, 49999098630, 51991000533, 54569573200, 54868526100, 59762285801, 60687013211, 60687013221, 63629126201, 68382038306, 69097031602 |

**Supplementary Table 2: Description of Variables Used in the Study**

| **Variable Name** | **Definition** |
| --- | --- |
| Telehealth utilization | Dummy variable: indicates telehealth use. |
|  | Continuous measure: number of telehealth visits. |
| Adherence status  (Outcome of interest) | Dummy variable: indicates adherent patients with Proportion of Days Covered (PDC) ≥80%. |
| Metastasis  (Outcome of interest) | Number of patients with an incident diagnosis of metastatic cancer during the follow-up period. |
| Direct costs  (Outcome of interest) | Continuous measure: total direct medical and pharmaceutical costs paid by patients for deductibles, copayments, and coinsurance. |
| Patient Age | Average age of the patient at first cancer diagnosis. |
|  | Ordinal variable: categorized into four groups: < 35, 35-44, 45-54, and 55-64. |
| Geographic region | Nominal variable: indicates whether patients were in the Northeast, North Central, South, or West. |
| Rurality status | Dummy variable: indicates patients not living in a Metropolitan Statistical Area (MSA value = zero). |
| Insurance plan type | Nominal variable: indicates insurance type (Preferred Provider Organization, Health Maintenance Organization, Consumer-Driven Health Plan, High-Deductible Health Plan, or other). |
| Type of endocrine agent | Nominal variable: indicates receipt of tamoxifen, a single aromatase inhibitor, or a switch from one agent to another. |
| CCI | Index of baseline health status: weighted scores assigned to each patient based on the number and severity of preexisting comorbid conditions. |
| CCI: Charlson Comorbidity Index | |

**Supplementary Table 3: Annual Summary of Telehealth Users and Visits**

| **Calendar Year** | **Telehealth Users (n)** | **Telehealth Visits (n)** |
| --- | --- | --- |
| 2018 | 37 | 210 |
| 2019 | 61 | 463 |
| 2020 | 627 | 2276 |
| 2021 | 465 | 2086 |
| 2022 | 414 | 2003 |
| 2023 | 296 | 1312 |
